# A123 CNON-SHERBROOKE NODE: ADVANCING COLORECTAL CANCER RESEARCH WITH ORGANOIDS

**DOI:** 10.1093/jcag/gwae059.123

**Published:** 2025-02-10

**Authors:** S Nassari, M Lecours, J Zhang, F Boudreau

**Affiliations:** Universite de Sherbrooke Faculte de Medecine et des Sciences de la Sante, Sherbrooke, QC, Canada; Universite de Sherbrooke Faculte de Medecine et des Sciences de la Sante, Sherbrooke, QC, Canada; Universite de Sherbrooke Faculte de Medecine et des Sciences de la Sante, Sherbrooke, QC, Canada; Universite de Sherbrooke Faculte de Medecine et des Sciences de la Sante, Sherbrooke, QC, Canada

## Abstract

**Background:**

Colorectal cancer (CRC) is a major global health issue and the second most common cancer in Canada. Human cancer cell lines have served as the main model for CRC research; however, they do not adequately capture the complexity and heterogeneity of tumors.

In the past decade, 3D in vitro models, known as organoids, have emerged as more accurate representations of organs’ structure and function. In cancer research, patient-derived organoids (PDOs) provide an ideal model that mimic the heterogeneity and clinical progression of the disease. However, fine-tuning this model needs numerous optimizations, which differ between laboratories and affect the reproducibility of the data. Additionally, accessing patient tissue for PDOs remains a significant challenge.

**Aims:**

The Canadian National Organoid Network (CNON), a multicentric (the University of British Colombia, University of Calgary & Université de Sherbrooke) national project supported by the Weston Family Foundation, was established to address these challenges. The Sherbrooke node is focused on building a biobank of CRC PDOs, using both tumor and healthy tissues from each patient to enable comparative studies. These PDOs, grown in various media, demonstrate differing growth patterns reflective of CRC heterogeneity.

**Methods:**

CNON Sherbrooke node, also focuses on optimizing advanced methodologies for human intestinal organoids to delve deeper into the biology of CRC and its clinical applications.

**Results:**

CRC arises from the accumulation of genetic mutations, yet the exact role of these mutations in cancer development remains unclear. To address this gap, we refine a comprehensive array of gene-editing tools, including CRISPR/Cas9, base editing, and prime editing, tailored for human intestinal organoids. This approach aims to produce organoids models that accurately reflect the genetic diversity and phenotypes associated with CRC, thereby enhancing the potential for personalized medicine.

Furthermore, the tumor microenvironment (TME) plays a crucial role in cancer progression, but its interactions with cancer cells and the immune system are poorly understood. To better simulate the TME, CNON-Sherbrooke node integrates organ-on-a-chip systems that combine multiple cell types. This innovative approach provides a better understanding of the TME’s involvement in the context of CRC for drug screening, and the development of precise therapies.

**Conclusions:**

In conclusion, the CNON-Sherbrooke node aims at first to enhance the accessibility of CRC-PDOs to the scientific community by optimizing and standardizing culture methods. Additionally, we seek to develop technological tools for CRC-PDOs. By combining organoids with genome editing and microfluidic systems, we aim to contribute to fundamental and translational research, drug discovery, and other CRC-related fields, ultimately enhancing the landscape of personalized medicine.

**Funding Agencies:**

The Weston Family Foundation, IRCUS (Institut de Recherche sur le Cancer de l’Université de Sherbrooke)

